# Functional aspects of salivary nitric oxide synthase of *Rhodnius prolixus* (Hemiptera, Reduviidae) and nitric oxide trafficking at the vector-host interface

**DOI:** 10.1038/s41598-017-16097-8

**Published:** 2017-11-22

**Authors:** Rafaela M. M. Paim, Bruno W. L. Nascimento, Ana Mércia D. Nascimento, Dimitri E. Pacheco, Adriana C. Soares, Ricardo N. Araujo, Mauricio R. V. Sant’Anna, Grasielle C. D. Pessoa, Nelder F. Gontijo, Marcos H. Pereira

**Affiliations:** Instituto de Ciências Biológicas, Universidade Federal de Minas Gerais, Belo Horizonte, MG Brazil

## Abstract

*Rhodnius prolixus* expresses nitric oxide synthase (NOS) in the cytosol of the salivary gland (SG) cells. The NO produced is stored in the SG lumen bound to NO-carrier haemeproteins called nitrophorins (NPs). NPs bind tightly to NO in the acidic SG lumen, but release NO when the pH becomes high, e.g., at the host skin (pH~7.4). NO elicits potent and transient relaxation of vascular smooth muscle. Here, we investigated the role of salivary NO in the *R*. *prolixus* feeding behaviour and the salivary vasodilator activity of the host microcirculation. NOS knockdown in *R*. *prolixus* changed the SG colour, decreased the number of NO-loaded NPs and caused impairment of feeding performance. When salivary gland extracts (SGEs) were obtained from NOS- and NPs-knockdown insects and prepared in pH 5.0 solution and injected (i.v.) into mice via the tail vein, no vasodilation was observed, whereas SGEs from control insects caused long-term venodilation in the mouse skin. SGs disrupted directly in PBS (pH 7.4) containing BSA produced long-term vasodilation compared to the controls without BSA due to the possible formation of nitroso-albumin, suggesting that host serum albumin extends the NO half-life when NO is injected into the host skin by triatomine during their blood-feeding.

## Introduction

The triatomine *Rhodnius prolixus* feeds on blood during all postembryonic stages. In addition to being an important vector of the protozoan parasite *Trypanosoma cruzi*, the etiological agent of Chagas’ disease, *R*. *prolixus* has been widely studied as a classical insect model. Knowledge about the physiology and biology of this insect has been improved by the large amount of available genetic information derived from transcriptomes, including the sialome^1^ and the recently finalised genome sequence^2^.

The arthropod’s salivary gland is a key organ that enables some of these organisms to develop a haemathophagous habit. Saliva provides a wide and redundant repertoire of biomolecules with anti-haemostatic activities, such as anticoagulants, platelet aggregation inhibitors and vasodilators, which are capable of facilitating the blood meal^3^. Examination of *R*. *prolixus* feeding on live hosts using labelled saliva have shown that saliva is continuously released into the host’s skin during the probing phase, whereas during the engorgement phase, saliva is released in low-frequency pulses (0.51 ± 0.2 Hz) inside the blood vessels, reinforcing the importance of the kissing bug’s salivary biomolecules throughout the blood-feeding process^4,5^.

Among several molecules already identified in *R*. *prolixus* and in the bed bug *Cimex lectularius* saliva, nitric oxide (NO) is particularly important due to its vasodilator and antiplatelet activities^6^. NO is extremely reactive and an unstable free-radical gas that can cross cell membranes to act on the surrounding environment^7^. The NO vasodilator and antiplatelet activities are mediated through activation of guanylate cyclase in endothelial and platelets cells, respectively^8^. NO production in the salivary glands is catalysed by the enzyme nitric oxide synthase (NOS), which uses L-arginine and molecular oxygen as substrates and NADPH as the co-factor^9–11^. In vertebrates, three isoforms of NO synthase have already been described: the neuronal nNOS (or NOS I), inducible iNOS (or NOS II), and endothelial eNOS (or NOS III). In insects, different isoforms have not yet been detected, and NOS seems to be homologous to the constitutive Ca^2+^-dependent NOS forms of invertebrates (nNOS and eNOS)^9^.

NO is not easily stored since it possesses a short half-life in biological systems due to its instability. In the *R*. *prolixus* salivary glands (SG), nitric oxide is reversibly bound to lipocalins called nitrophorins (NPs), which correspond to an abundant group of haemeproteins responsible for conferring the typical reddish colour of the SGs. NPs are NO-carrying haemeproteins that tightly bind NO in the low-pH environment of the salivary gland lumen (pH ≤ 6) and release NO when the pH increases, as occurs when saliva is secreted in the host skin, in which the pH is approximately 7.4^9^. In mammalian hosts, it was demonstrated that active thiols (cysteine residues with free lateral chains) is abundantly distributed in plasma and are able to incorporate and transport NO as *S*-nitrosothiols^12^. S-nitroso-albumin is the major *S*-nitrosothiol that circulates in plasma and probably acts as an NO reservoir, slowly releasing NO as the thiol concentration increases^13,14^.

Although NO causes transient relaxation of vascular smooth muscle, preliminary observations of mouse skin microcirculation have shown persistent vasodilation after a *R*. *prolixus* blood meal. This observation allows the possibility that host proteins interact with the NO released from the insect saliva at the bite site to ensure a more persistent vasodilatation and, therefore, a more successful blood meal.

In this study, we evaluated the NOS gene expression levels in the *R*. *prolixus* salivary glands and reduced NO gene expression to address the role of NO in the *R*. *prolixus* feeding performance and NO vasodilation when it is ejected from the insect salivary glands into the microcirculation of the host skin.

## Results

### Evaluation of NOS expression in different organs

**Figure 1:**
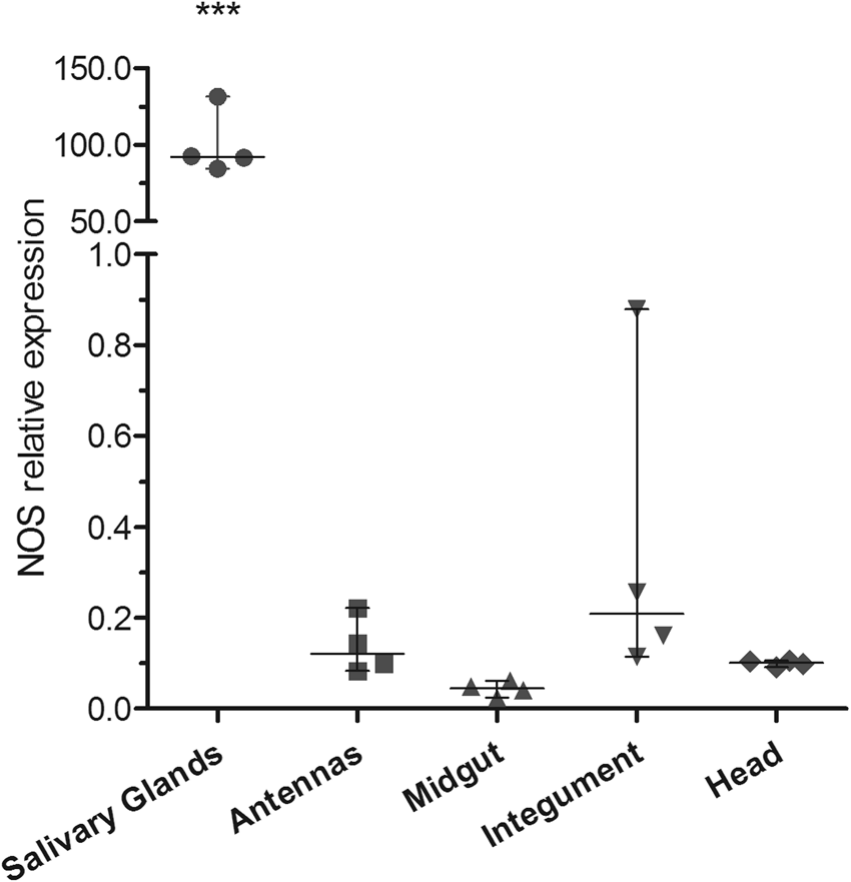
qPCR expression of the NOS gene in different organs of the *R*. *prolixus* 5^th^ instar nymphs. Data are represented by the median ± range of 4 pools of 3 organs. ***ANOVA, Bonferroni’s test, p < 0.001, compared to other groups.

The NOS expression profile was determined by qPCR for different organs of the 20-day-starved *R*. *prolixus* 5^th^ instar nymphs. In the salivary glands (SGs), NOS expression was much higher compared to the other organs (antenna, midgut, integument and head), especially in comparison to the midgut, which contained 2000-fold fewer NOS transcripts in comparison to the SGs (Fig. 1).

### Expression of NOS and nitrophorin 2 in the salivary glands after blood feeding

**Figure 2:**
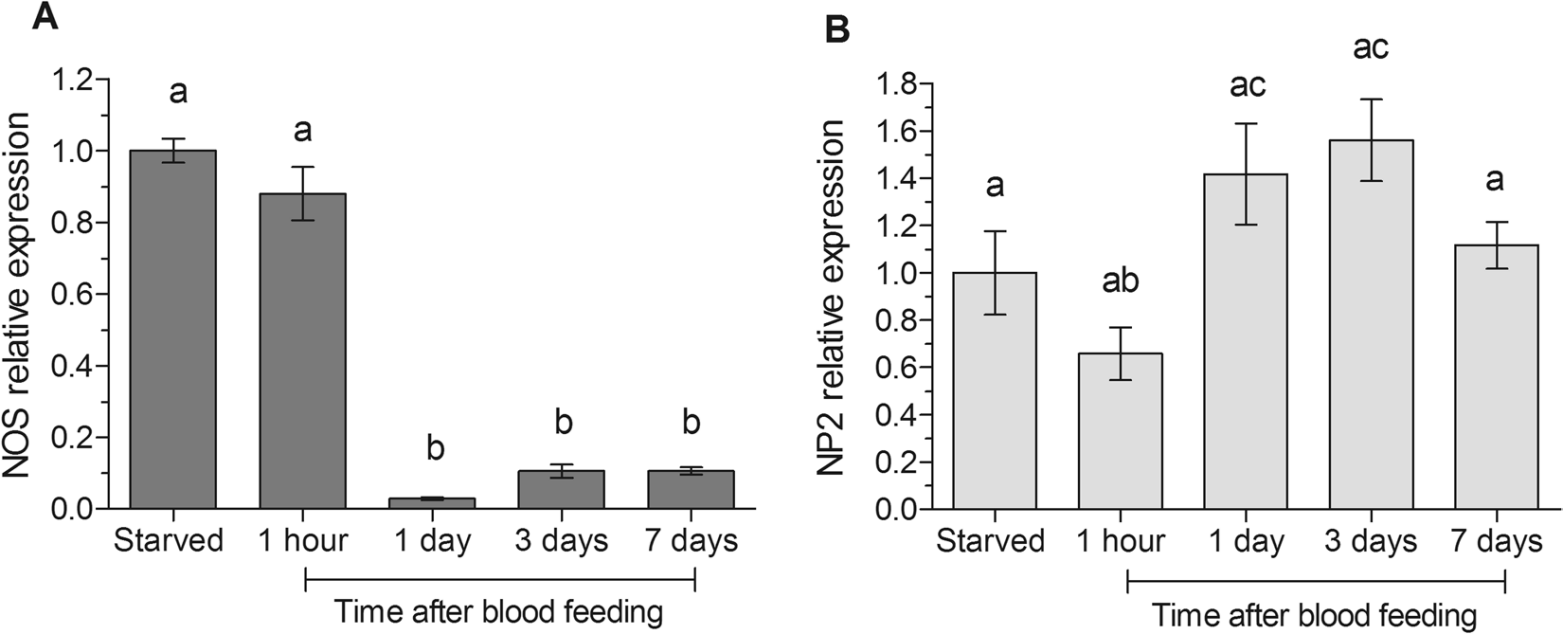
qPCR analysis of the expression levels of (**A**) nitric oxide synthase (NOS) and (**B**) nitrophorin 2 (NP2) genes in the salivary glands of the *R*. *prolixus* 5^th^ instar nymphs during starvation (20 days) and after blood feeding (1 hour, 1 day, 3 days and 7 days). Data are represented by the mean ± SEM of 5 pools of three salivary gland pairs for each point. Different letters indicate statistically significant differences (ANOVA, Bonferroni’s test, p < 0.05).

The gene expression patterns of the enzyme responsible for producing NO (NOS) and one of its carrier proteins, nitrophorin 2 (NP2), were evaluated in the SGs of 5^th^ instar nymphs, under starvation (20 days of starvation after moult) and after blood feeding (1 hour, 1 day, 3 days and 7 days). Under starvation and soon after blood feeding, NOS presented a high expression, which significantly decreased from day 1 and remained low until the day 7 after feeding (Fig. 2A). On the other hand, NP2 expression is relatively constant throughout the evaluated period, presenting a lower expression only at 1 hour after feeding (Fig. 2B).

### Confirmation of RNAi knockdown of NOS in *R*. *prolixus* salivary glands

**Figure 3:**
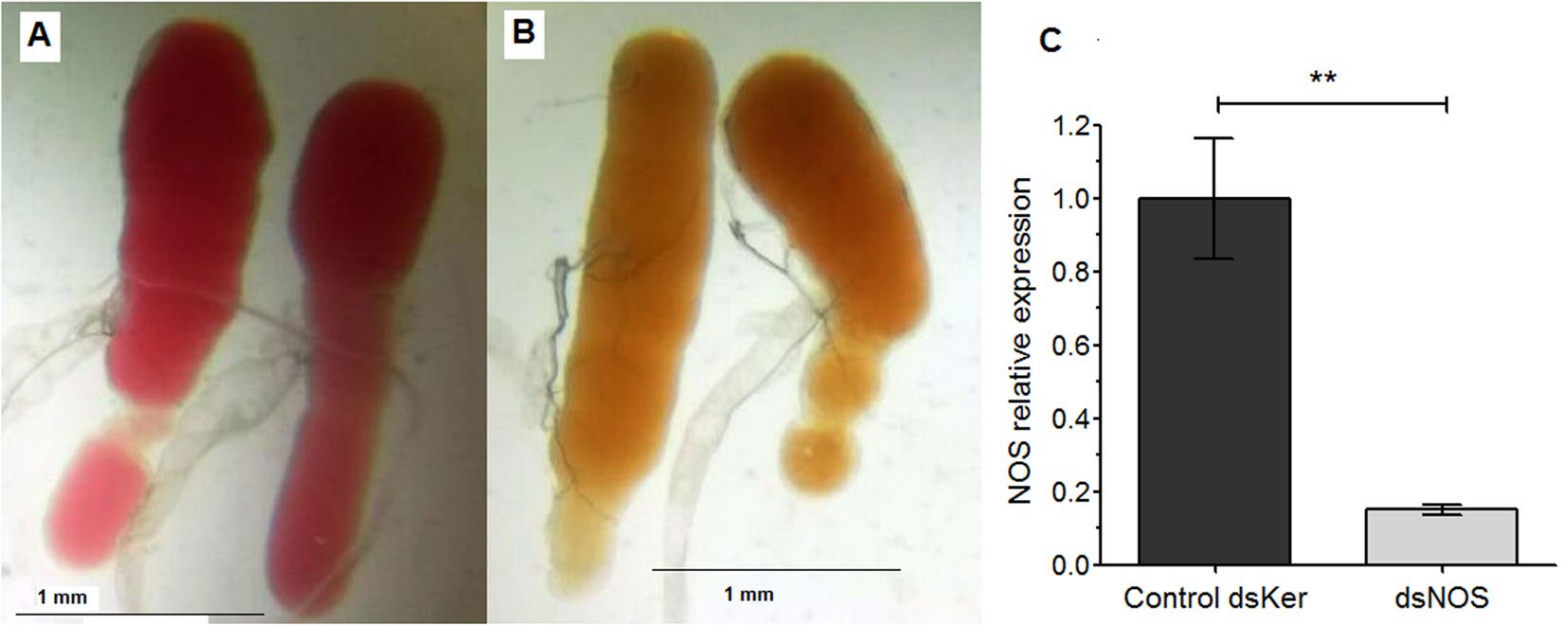
Evaluation of NOS knockdown in the salivary glands of 5^th^ instar nymphs injected with 5 µg of dsRNA (dsKer or dsNOS) during the third instar. The salivary glands of the (**A**) control dsKer insects and (**B**) dsNOS knockdown insects show their reddish and brownish colours, respectively. (**C**) The NOS mRNA levels in the dsKer- and dsNOS-injected insects were evaluated by qPCR. Data are represented by mean ± SEM of six pools of three salivary gland pairs per group. **Mann-Whitney, p < 0.01.

To obtain NOS knockdown insects, 5 µg of dsNOS were injected into the third instar nymphs, and knockdown confirmation was assessed in the 5^th^ instar. At this time, an 85% significant reduction in the NOS mRNA levels was observed (Fig. 3C, Mann-Whitney, p < 0.01). The dsKer-control insects had salivary glands with the typical reddish colour (Fig. 3A), whereas the NOS knockdown SGs showed an altered brownish colour (Fig. 3B).

**Figure 4:**
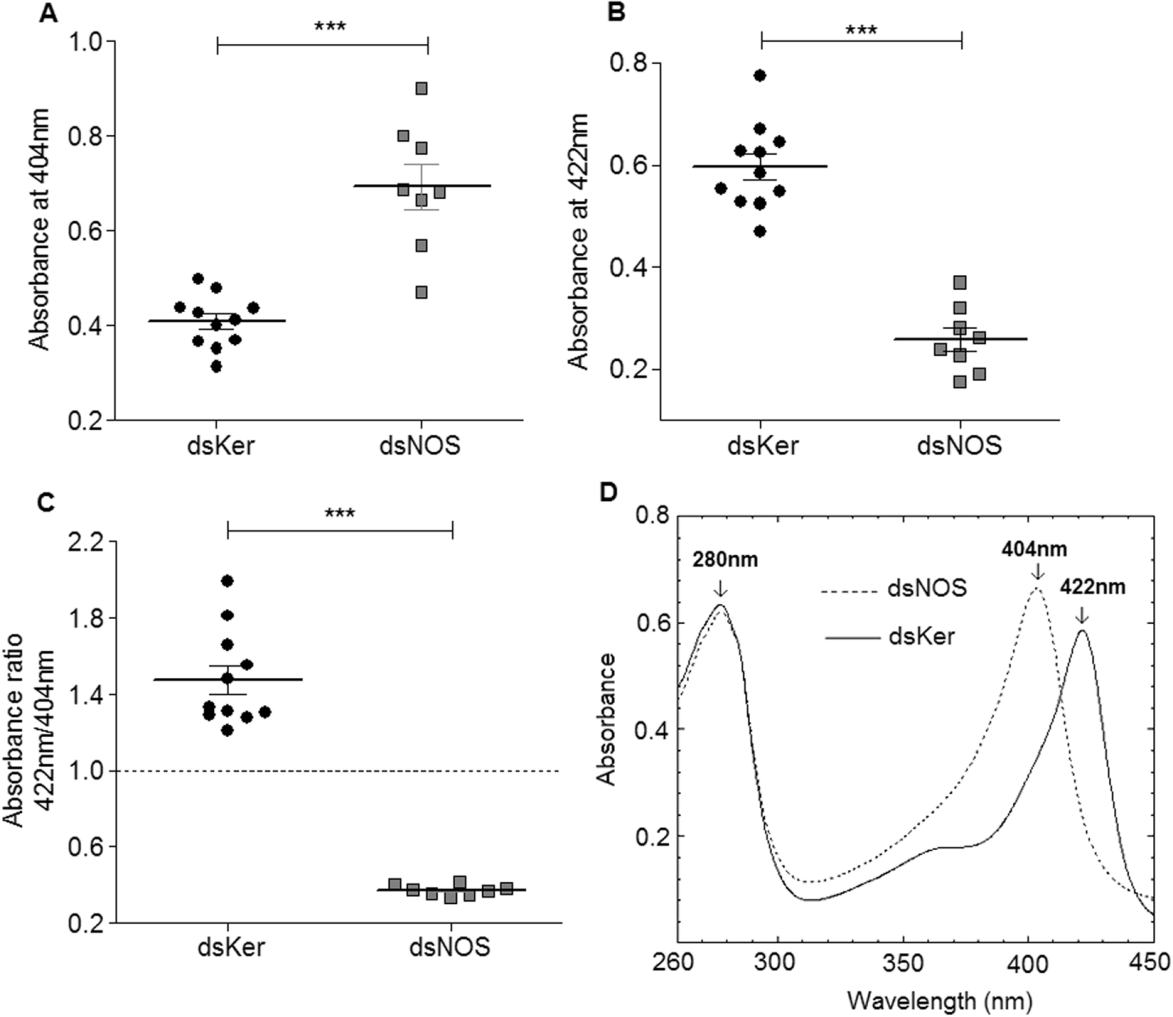
Phenotype evaluation of the NOS knockdown insects by spectrophotometric assessments of nitrophorins (**A**) with (404 nm) or without (422 nm) (**B**) nitric oxide. (**C**) The absorbance ratio at 422 nm/ 404 nm was used to estimate the degree of NO loading of the nitrophorins. (**D**) Typical spectrophotometric profiles of the dsKer SGE (peak at 422 nm) and dsNOS SGE (peak at 404 nm). The individual salivary glands (SGs) of the *R*. *prolixus* 5^th^ instar nymphs were dissected and disrupted in AcB-pH 5.0. At this pH, most nitrophorins remained loaded with NO. Data are represented by mean ± SEM of salivary gland extract pairs of the control insects injected with dsKer (n = 11) or the dsNOS knockdown insects (n = 8). ***Student t-test, p < 0.001.

The NOS knockdown phenotype was also evaluated by estimating the level of nitric oxide production, which was indirectly assessed using the spectrophotometric profiles of the SGs disrupted and maintained in sodium acetate buffer pH 5.0 (AcB-pH 5.0). The total amount of haemeprotein was similar between the dsKer- and dsNOS-injected insects (Student t-test, n.s), as assessed using the sum of the absorbances at 422 nm and 404 nm. The salivary gland extracts (SGEs) of the dsKer insects showed higher absorbance values at 422 nm than the dsNOS insects (Fig. 4B), which indicated higher amount of NO-bound nitrophorins, whereas in the dsNOS insects, higher absorbances were observed at 404 nm (Fig. 4A), indicating that most nitrophorins were not bound by NO. The degree of NO-loaded nitrophorins was estimated using the ratio of the absorbances at 422 nm/404 nm (Fig. 4C). In the dsKer-control insects, the rate of NO-loaded nitrophorins was 4-fold higher than in the NOS knockdown insects, indicating a significant reduction in NO production in this group (Student t-test, p < 0.001). These results were confirmed using the spectral profiles of the SGE, in which a common peak was observed at 280 nm (related to total protein); however, the dsKer insect group showed a distinctive peak at 422 nm, whereas the insects from the dsNOS group showed a peak at 404 nm (Fig. 4D).

### Effect of NOS knockdown on the feeding performance of *R*. *prolixus* on anaesthetised rats

**Figure 5:**
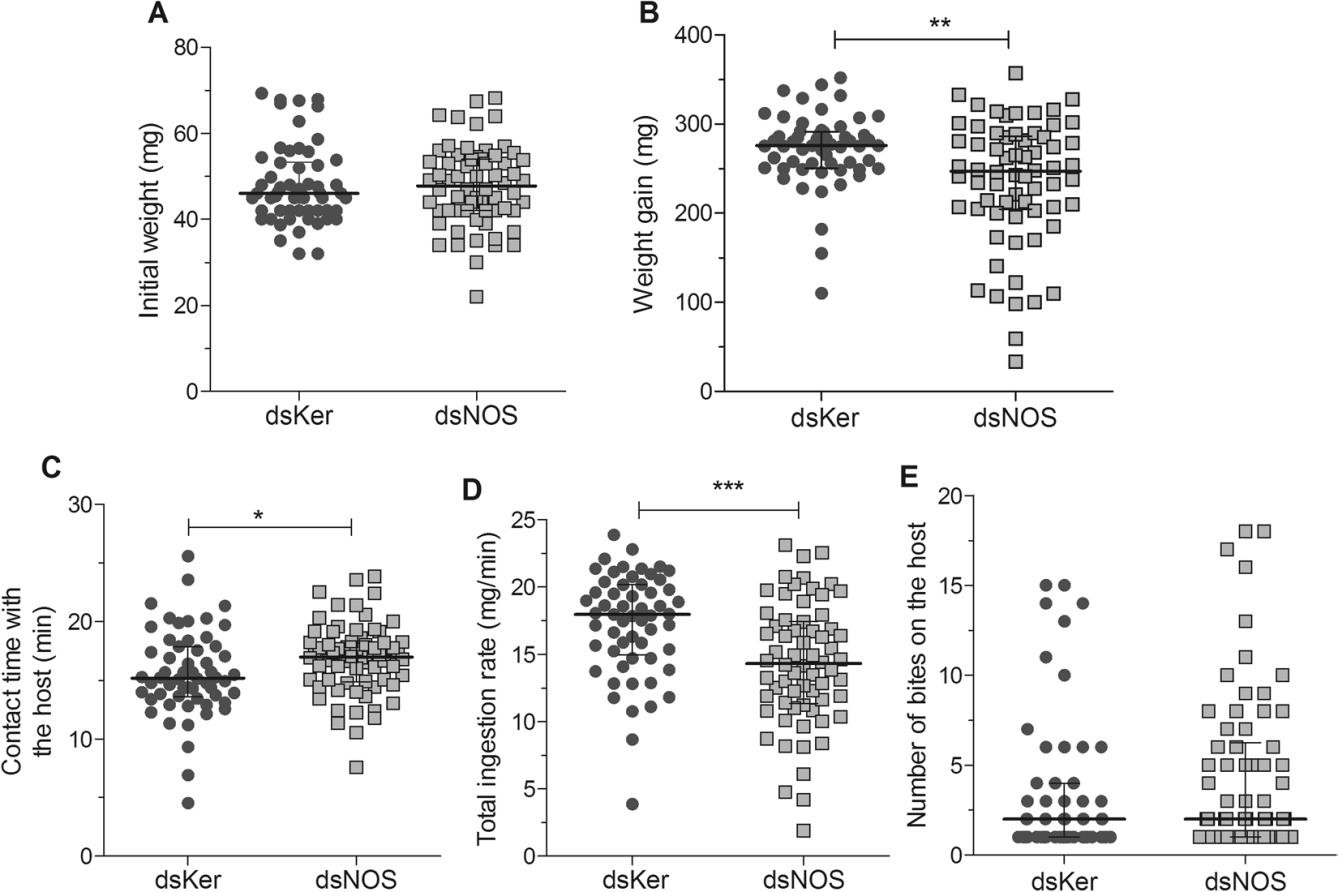
Feeding performance parameters of *R*. *prolixus* 5^th^ instar nymphs injected with dsKer (n = 58) or dsNOS (n = 64) that fed on the dorsal surfaces of anaesthetised rats. All blood feeds were recorded, and each insect was weighed (mg) before (**A**) and immediately after (**B**) the blood meal to measure weight gain (mg). The total contact time of the insect with the host (min) (**C**) was also measured to determine the total ingestion rate (mg/min) (**D**). The number of bites on the host (**E**). Data are represented by the median ± interquartile range. Mann-Whitney, *p < 0.05, **p < 0.01, ***p < 0.001.

To assess the role of NOS in the feeding performance of *R*. *prolixus*, the behaviours of 5^th^ instar nymphs on the rat dorsal surfaces were monitored. Before feeding, the nymphs of both groups showed similar initial weights (Fig. 5A, Mann-Whitney, p > 0.05). The insects injected with dsNOS ingested significantly lower amounts of blood compared to the control group (Fig. 5B) and demanded more contact time with the host to complete their blood meals (Fig. 5C) (Mann-Whitney, p < 0.05). These parameters impacted the total ingestion rate (mean ± SD), which was 17.26 ± 3.8 mg/min for the dsKer insects and 14.29 ± 4.5 for the dsNOS insects, indicating that a lack of NO in the SGs notably affected the feeding efficiency of these bugs (Fig. 5D, Mann-Whitney, p < 0.001). Although the dsNOS insects inflicted a slightly higher number of bites on the host before starting the blood intake, this difference was not significant compared to the control (dsKer) insects (Fig. 5E, Mann-Whitney, p > 0.05). The degree of NOS knockdown in each insect was confirmed after feeding by removal and visualisation of the salivary gland colour (brownish colour), compared to the controls (reddish colour) (Fig. 3A and B).

### Vasodilator activity

#### Intravenous injections

**Figure 6:**
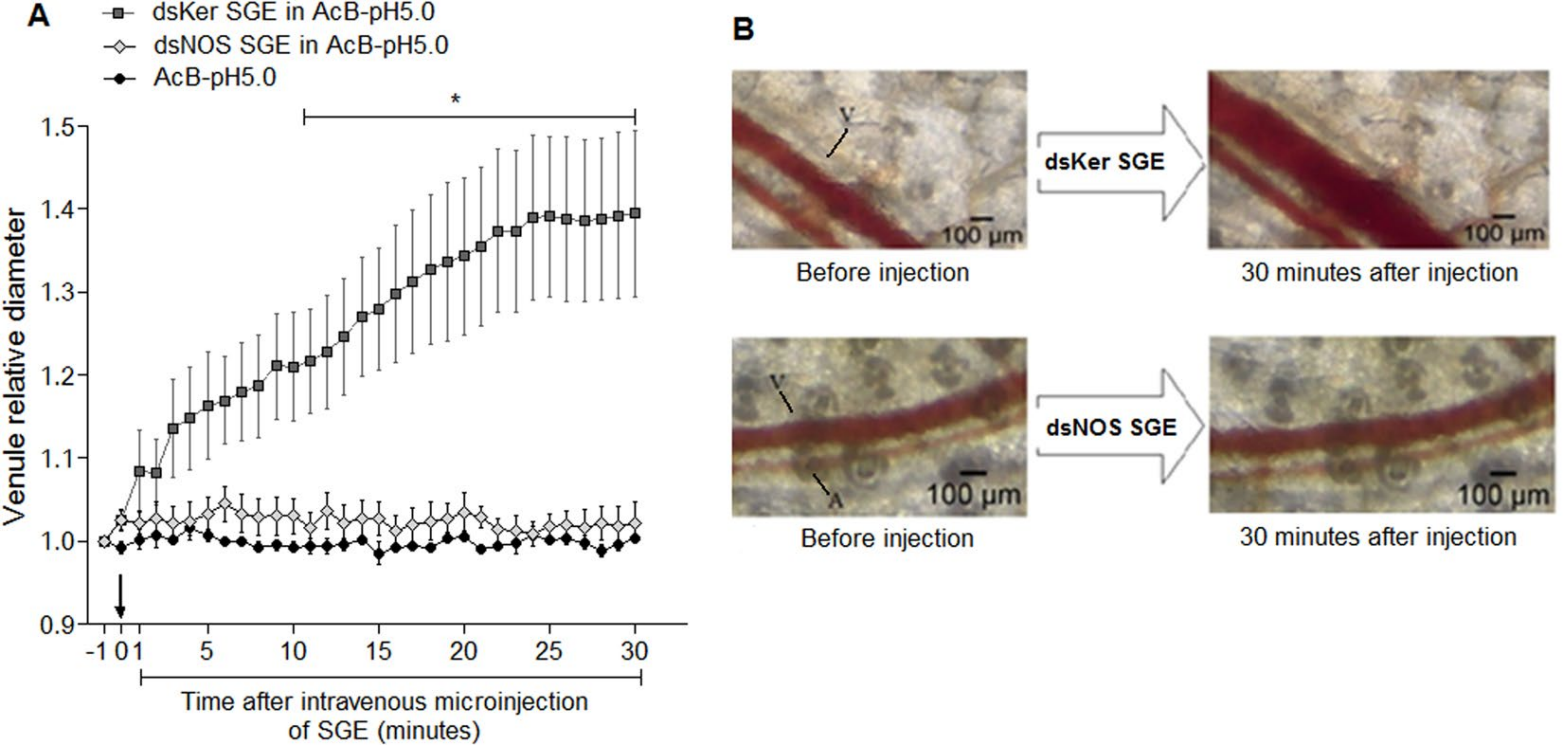
Alterations in mouse ear microcirculation throughout the time after the tail-vein intravenous injections of the mice with one salivary gland pair extract (SGE) obtained by SG disruption in 70 µL AcB-pH 5.0 (10 mM sodium acetate and 150 mM NaCl, pH 5.0) of *R*. *prolixus* injected with keratin dsRNA (dsKer) or nitric oxide synthase dsRNA (dsNOS). (**A**) Relative venule diameter throughout the first 30 min after the intravenous injection. The venule diameter value before the injection was arbitrarily set to 1 to allow arelative comparison. On the X axis, -1 corresponds to the moment before the SGE injection, and 0 corresponds to the moment immediately after the SGE injection (black arrow). Data are represented by mean ± standard error (n = 5 for each time point). The analysis was performed using two-way repeated measures ANOVA and the Bonferroni’s test, *p < 0.05. (**B**) Representative microscopic image of the microcirculation in the mouse ear before and 30 min after the intravenous injection. *A*, arteriole and *V*, venule.

The SGEs of the dsKer- and dsNOS-injected insects were intravenously inoculated into the tail vein of mice to observe alterations in the microcirculation of the mouse ear by intravital microscopy. To maintain NO association with nitrophorins, the SGs were disrupted directly in AcB-pH 5.0, the same solution used for spectrophotometric estimations of NO-loaded nitrophorins (Fig. 4). In this condition, a single SG pair from the dsKer-injected insects was able to induce vasodilation of the ear venules as early as 1 minute after the injection. The dilation increased overtime, and at 30 min after the intravenous injection, the venule diameter was approximately 40% higher than the baseline (Fig. 6A and B). On the other hand, the venule diameter did not significantly change during the first 30 min after the dsNOS SGE injection (Fig. 6A and B). Beginning at the 11^th^ min after the intravenous injections, the dsKer SGEs induced a significant increase in the venule diameter (two-way ANOVA, Bonferroni’s test, p < 0.05), in comparison to the dsNOS SGEs and negative control (injection of AcB-pH 5.0 solution only), which showed similar venule dilation profiles throughout the first 30 min after the injections (Fig. 6A).

**Figure 7:**
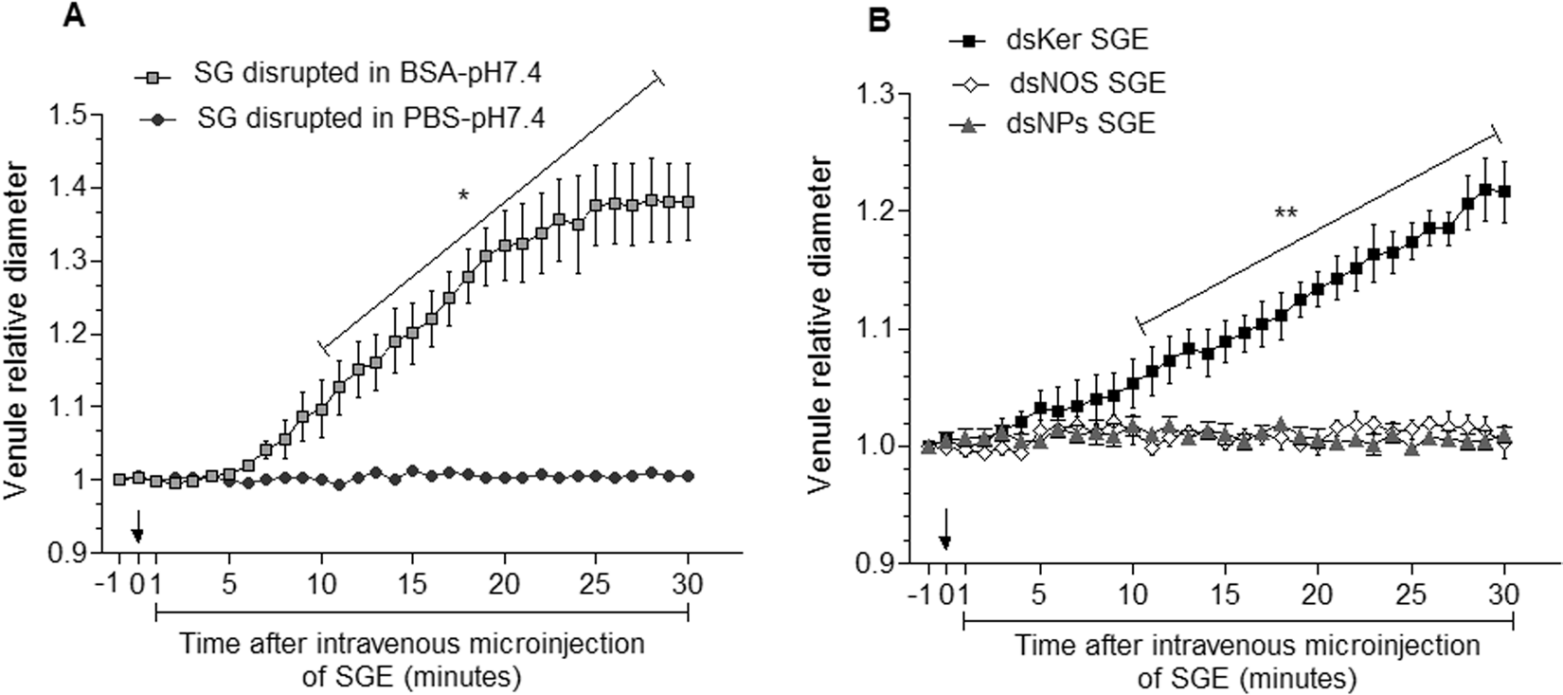
Alterations in the diameter of the mouse ear skin venules throughout the time period after tail-vein intravenous microinjections with *R*. *prolixus* salivary gland extracts (SGEs), which were obtained by disruption of three salivary gland (SG) pairs in a PBS-pH 7.4 solution or in the presence of bovine serum albumin (BSA-pH 7.4 solution) (**A**), or using *R*. *prolixus* SG extracts disrupted in a BSA-pH 7.4 solution from insects injected with 5 µg keratin dsRNA (dsKer), nitric oxide synthase dsRNA (dsNOS) or nitrophorins dsRNA (dsNPs). The venule diameter before the injection was arbitrarily set to 1 to allow a relative comparison. On the X axis, -1 corresponds to the moment before the SGE injection, and 0 corresponds to the moment immediately after the SGE injection (black arrows). Data are represented by the mean ± standard error (n = 5). Statistical analysis was performed using two-way repeated measures ANOVA, *Bonferroni’s test, p < 0.05, **Bonferroni’s test, p < 0.01.

The long-lasting vasodilation throughout the first 30 min after the SGE inoculation via intravenous injection suggested that NO might be interacting with a host molecule, increasing its half-life and extending its action. To investigate whether this molecule was the host serum albumin, we tested the injection of *R*. *prolixus* SGE obtained by SG disruption in the presence of bovine serum albumin (BSA). SGs that were disrupted using only the PBS-pH 7.4 solution did not alter the diameters of the mouse ear venules. Conversely, a robust and significant vasodilation was observed beginning at the 11^th^ min after the intravenous injections of the SGs disrupted in the BSA-pH 7.4 solution (two-way ANOVA, Bonferroni’s test, p < 0.05) (Fig. 7A). Based on this experiment, which showed that vasodilation could be triggered by SG disruption in the presence of BSA (Fig. 7A), we tested the vasodilation activity of the SGEs in BSA-pH 7.4 of the dsKer, dsNOS and dsNPs insects. Confirmation of the NPs knockdown was easily performed by observing the loss of the SG reddish colour (SG became transparent upon nitrophorins knockdown), according to Araujo *et al*.^15,16^. While the dsKer SGEs induced significant vasodilation in the ear venules beginning at the 11^th^ min after the intravenous microinjection (two-way ANOVA, Bonferroni’s test, p < 0.01), the dsNOS and dsNPs SGEs were not able to promote a significant increase in the venule diameter during the first 30 min after the intravenous injections (Fig. 7B). The SG extracts of the dsKer insects promoted an increase of 21% in the diameters of the mouse ear venules after 30 min (Fig. 7B).

### Intradermal injections

**Figure 8:**
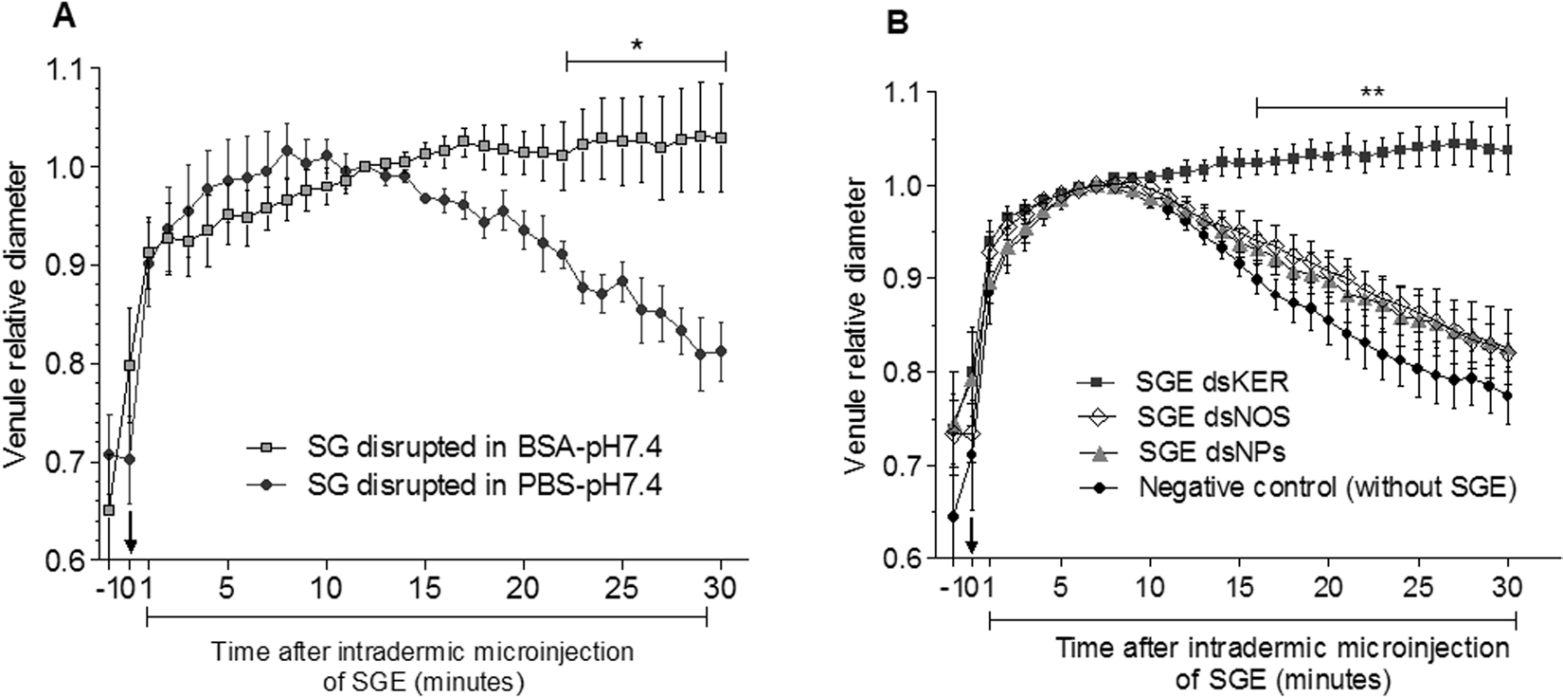
Alterations in the diameter of the mouse ear skin venule over time after the ear intradermal microinjections of 32.2 nL of *R*. *prolixus* salivary gland extracts (SGEs), which were obtained by (**A**) salivary gland (SG) disruption in PBS-pH 7.4 solution or disruption in the presence of albumin (BSA-pH 7.4 solution). (**B**) Activity levels of the SGEs from the *R*. *prolixus* injected with keratin dsRNA (dsKer), nitrophorins dsRNA (dsNPs) or nitric oxide synthase dsRNA (dsNOS) that were disrupted in the BSA-pH 7.4 solution. In the negative control, only the BSA-pH 7.4 solution (without the SGE) was injected. The values of maximum dilation were arbitrarily set to 1 to allow a relative comparison in each experiment. On the X axis, -1 corresponds to the moment before the SGE injection, and 0 corresponds to the moment immediately after the SGE injection (black arrows). Data are represented by the mean ± standard error (n = 5 for each time point). The analysis was performed using two-way repeated measures ANOVA and Bonferroni’s test, *p < 0.05, **p < 0.01.

Next, we evaluated the intradermal SGE microinjections directly in the mouse ear, comparing the SGs disrupted in the PBS-pH 7.4 solution with the SGs disrupted in the presence of albumin (BSA-pH 7.4 solution). Through the intradermal route, the *R*. *prolixus* SGEs induced an immediate and robust vasodilation response during the first minute after the injection, regardless of the medium in which the SGs were disrupted (Fig. 8A). Afterwards, the extracts obtained by SG disruption in the PBS-pH 7.4 solution promoted a significant reduction in the venule diameter from the 22^nd^ until the 30^th^ min after the injection (two-way repeated measures ANOVA, Bonferroni’s test, p < 0.05), (Fig. 8A). On the other hand, the SGs disrupted in the BSA-pH 7.4 solution were able to maintain venule dilatation at high levels until the 30^th^ min (Fig. 8A).

Using only SGEs in the BSA-pH 7.4 solution, we also evaluated the effects of NOS and NPs knockdown using intradermal injections. In this condition, the SGEs from the dsKer insects triggered a significant increase in venule diameter from the 16^th^ until the 30^th^ min after the microinjection (two-way repeated measures ANOVA, Bonferroni’s test, p < 0.05) (Fig. 8B). The SGEs from both knockdown insects (injected with dsNOS or dsNPs) produced similar responses in the venule diameter, which was reduced over time after the initial vasodilation up to the 30^th^ day; this pattern was also very similar to that observed for the negative control (injection of only BSA-pH 7.4 solution, without SGEs) (Fig. 8B).

## Discussion

NOS expression was observed in multiple organs suggesting that NO may perform different functions in various biological processes. In triatomines, NOS activity was first reported in SGs^9^ but was later described in different tissues^17^. NO activity have been associated with insect immune responses against pathogens, which can stimulate NOS expression in haemocytes and other insect organs^17,18^. NO can also act as a signalling molecule, and its production has also been reported in the central nervous system of *Triatoma infestans* ^19^. Sfara *et al*.^20,21^ discussed a possible role for NO in the *R*. *prolixus* olfactory pathway and in the repellent perception and response.

NOS expression was detected in all evaluated organs (SG, antenna, midgut, carcass and head), and the NOS sequence used in this study showed much higher expression in the salivary glands compared to all other organs, which indicates the importance of NOS in the SG. According to Yuda *et al*.^10^, *R*. *prolixus* SG cells express huge amounts of NOS that was not observed in any other animal cells. Other works had reported that NOS enzymatic activity can be regulated by multiple and different factors^22^. Therefore, sometimes it is difficult to positively correlate NOS expression to NO activity^19,23^. Perhaps this can help to explain why we did not find higher levels of NOS transcripts in the insect’s head in comparison to midgut and integument, considering that NO have already been described as an important signaling molecule in insect’s central nervous system, including triatomines^19^. The NOS activity described in triatomines is NADPH-, FAD-, calmodulin- and Ca^+2^-dependent. From these characteristics, *R*. *prolixus* and NOS from other invertebrates are very similar to the constitutive NOS from vertebrates^9,10^. To date, it is not known if there are multiple NOS isoforms in insects and if their multiple functions are related to the same or different enzymes^24,25^.

NO-carrier haemeproteins (nitrophorins) are exclusively present in haematophagous reduviids of the Rhodniini tribe^26–28^. Although all four nitrophorins (NPs1-4) have been previously knocked down by RNAi, we chose nitrophorin 2 (NP2) to represent the expression of the nitrophorins family, as it is expressed at high levels in all *R*. *prolixus* stages^29^. All nitrophorins are highly conserved lipocalins found in *Rhodnius* salivary glands.

In the present work, NOS and NP2 molecules exhibited distinct expression profiles. NP2 is produced and secreted to the gland lumen, where it is stored until its release with the insect’s saliva during the blood meal. The NP2 expression profile suggests that this molecule was constantly produced in the evaluated period, accumulating in the salivary glands between blood feeds. On the other hand, NOS is an intracellular enzyme that is produced before feeding, and only its product (NO) is secreted into the gland lumen; the enzyme remains active in cells even after feeding. Our results validated the findings of Gazos-Lopes *et al*.^24^, which used semi-quantitative PCR and western blot to demonstrate that NOS expression in salivary glands was high immediately after feeding but decreased over the first days after feeding, increasing again only thirteen days after feeding, when the insect was close to undergoing ecdysis. NPs are already synthesised when NO starts to be intracellularly produced, and afterwards, NO is probably exported to the gland lumen, where it binds to the NPs^30^.

As with the NP knockdown, which caused the *Rhodnius* SG to become virtually transparent^16,31^, we observed that the NOS knockdown also markedly altered the SG colour. *R*. *prolixus* SGs typically lose their cherry red colour in the absence of the haemeproteins that confer the typical reddish colour or when the haemeproteins are present but not bound to NO. These phenotype alterations were very useful for confirming gene knockdown in the insect before the SGE injections into the mouse ear or after the feeding performance experiments.

An alternative method for evaluating the phenotypic effects of NOS knockdown is to indirectly evaluate the lack of NO in the SG by the quantification of NO-loaded or NO-free nitrophorins. This is possible because nitrophorins display different absorption peaks when is NO-loaded (422 nm) and in the absence of NO (404 nm)^30^. In this context, impairment of NO synthesis by NOS knockdown can be easily detected by the increase in the absorption at 404 nm and reduction in the absorption at 422 nm, considering that total amount of haemeproteins (404 nm + 422 nm) is very similar when the control and NOS knockdown insects are compared. For this assay, disruption and maintenance of SGs in an acidic buffer is essential to maintain active NP-NO binding, and the sodium acetate buffer (pH 5) used here for this purpose have been previously used for *Rhodnius* ^30^ and for *Cimex* ^32^.

In the present study, we observed that NOS knockdown insects exhibited a reduced feeding efficiency compared to the control, which was consistent with the observations of Araujo *et al*.^16^ for NPs knockdown bugs, which presented lower ingestion rates than control insects. The differences observed for some feeding parameters (weight gain and contact time) are more related to methodological differences between these two studies. In the current study, the feeding of control and NOS knockdown insects on the dorsal surfaces of anaesthetised rats was limited to a maximum of 25 min. After this time, the feeding was interrupted even if the insects were not completely engorged. In this context, NOS knockdown insects spent more time in contact with the host and ingested smaller amounts of blood, which had a significant effect on the total ingestion rate. Impairment in the feeding process was observed in both the NPs knockdown^16^ and NOS knockdown insects here, but the NPs knockdown seemed to have affected the *R*. *prolixus* feeding performance more intensely, probably due to the multiple functions that were affected by NPs suppression. On the other hand, the NOS knockdown mostly affected the vasodilation response. In addition to being NO-carrier molecules, NPs have many other functions, and their depletion in SGs also affects their antihistaminic activity, which is intended to prevent swelling and pain and therefore allow insects to avoid being detected by the host^33^ and the anticoagulant activity, which is induced by NP2. In the present study, depletion of salivary NO specifically affected the insect’s ability to induce vasodilation and inhibit platelet aggregation at the bite site. Our results demonstrated the importance of these two anti-haemostatic activities to ensure an efficient blood meal for *R*. *prolixus* bugs. In insects, vasodilator molecules act in the blood feeding process by increasing the host blood vessel diameter, which reduces the probing time and enhances blood flow at the bite site, reducing the feeding time of the insect^34^. Despite the role of NO in platelet aggregation inhibition, *R*. *prolixus* has other important molecules that are able to perform this function in the SGs, such as apyrases^35,36^.

In the present study, we confirmed that NO was the main molecule responsible for the vasodilator activity of the *R*. *prolixus* saliva. The decrease in NO production by NOS knockdown significantly affected the competence of the saliva to induce vasodilation in the host microcirculation, regardless of how the saliva was introduced into the host (intravenous or intradermal injections). In standard SG physiological conditions (pH ≤ 6), nitrophorins act as NO reservoirs and NO is promptly released when this carrier protein is exposed to the host bloodstream. The half-life of NO in biological fluids is very short, with an estimated half-life of in blood of just a few milliseconds^12^. The main products of immediate NO breakdown are nitrite and nitrate, which are not vasodilators. To efficiently perform its function, which is to induce relaxation of the vessel musculature under physiologic conditions, the released NO reacts with free thiol groups from proteins, forming S-nitroso-protein compounds such as S-nitroso-albumin, which is the predominant species circulating in plasma^14^. Our results using intravital microscopy confirmed this information and suggest that albumin, which circulates in abundance in mammalian serum, can interact with the NO released by the *R*. *prolixus* salivary nitrophorins at the bite site. Such an interaction can occur between the thiol group of the free-cysteine residue of serum albumin, forming S-nitroso-albumin in the host microcirculation, which would increase the half-life of NO through its slower and constant release into the host tissues and vessels, inducing vasodilation, as previously shown by Orie *et al*.^13^.

In this context, the possible formation of S-nitroso-albumin could explain the long duration of vasodilation observed in the mouse ear venules, which persisted for at least 30 minutes after the injection of SGE from the dsKer-control insects. It is expected that when the SGs of these insects were disrupted in the AcB-pH 5.0 solution, the acidic medium was able to retain NO until it was taken up by the hairless mouse albumin (S-nitroso-thiols), inducing a fast and robust vasodilation. In this condition, the increase in the venule diameter was already apparent within the first minute after the intravenous injection, reaching a 40% increase in diameter after 30 minutes (Fig. 6A). On the other hand, the SGE obtained in PBS pH 7.4 only promoted vasodilation when the SG was disrupted in the presence of bovine serum albumin. This condition seems to have been slightly less efficient because vasodilation began to appear only five minutes after the SGE intravenous injection and only a 20% increase in the venule diameter was observed after 30 minutes (Fig. 7B). Albumin is the major serum protein, and in addition to binding nitroso compounds, can carry a wide variety of fatty acids and other lipophilic compounds that have important roles in the regulation of blood osmotic pressure^37^.

Experiments using *R*. *prolixus* 5^th^ instar nymphs to feed on mouse skin (ear or dorsal surface) elicited long-term vasodilation in the ear (data not shown). To mimic the effect of an insect bite on the skin, we introduced the SGE via intradermal injection directly into the mouse ear skin. In the intradermal injections, regardless of the injected solution or the presence of the SGE, an immediate nonspecific and potent vasodilator response was detected in the ear vessels. This response is probably associated with neurogenic vasodilation due to stimulation of the sensory nerves during the mechanical introduction of the microinjector needle in the mouse skin. The occurrence of antidromic pulses, which are components of neurogenic inflammation, has already been described as a physiological mechanism to elicit cutaneous vasodilation^38,39^. Considering this situation, we analysed these experiments only after the venules had reached a maximum dilation value. From this moment, which occurred approximately 10 minutes after the injection, the venules diameters started to decrease, as observed for the SGs disrupted in the PBS-pH 7.4 solution (Fig. 8A) and for the SGs disrupted in the BSA-pH 7.4 solution and using SGs obtained from the knockdown insects (injected with dsNOS or dsNPs). In contrast, using the SGEs from the dsKer-control insects, the venule diameters continued to increase after neurogenic vasodilation, remaining at high levels over 30 minutes after the SGE injection (Fig. 8B). It is worth mentioning that this immediate neurogenic vasodilatation response is not observed after biting or even during the probing phase of *R*. *prolixus* feeding on the mouse ear^40^, probably due to less tissue damage produced and the presence of anaesthetic activity in the saliva of the insect, as demonstrated in other triatomine species^41,42^.

Using intravenous injections, a relaxation effect could be immediately observed (in the first minute post-injection) in the ear vessels of the animal. Under the experimental conditions (low concentration), the AcB-pH 5.0 solution was inert and did not induce any alteration in the vessel diameter.

**Figure 9:**
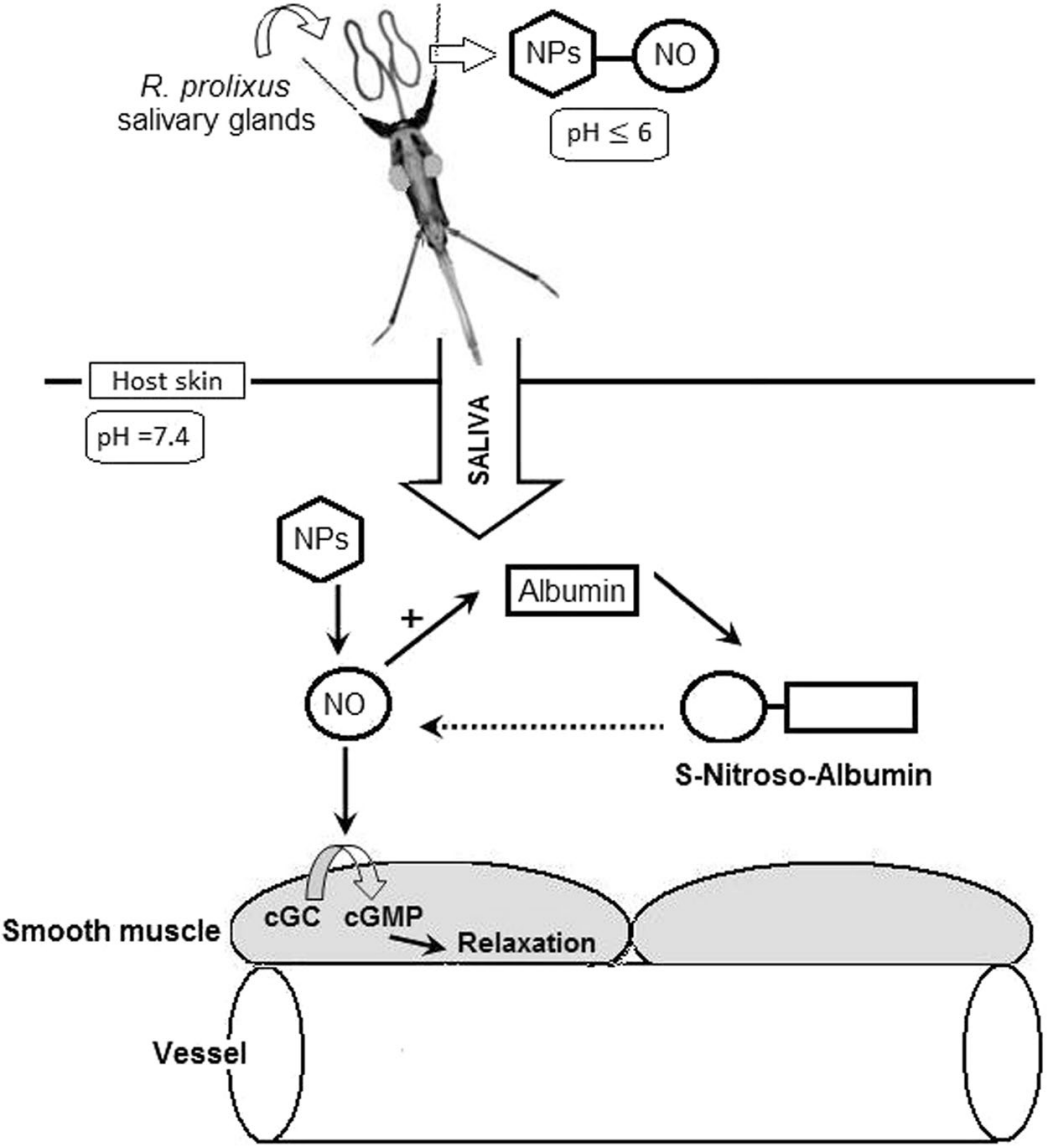
Representative scheme of nitric oxide (NO) release from *R*. *prolixus* nitrophorins (NPs) and the interaction with the vertebrate host molecules at the bite site, causing vasodilation. The solid black arrows represent fast events, and the dotted arrow represents slow events. *NO*, nitric oxide; *NPs*, nitrophorins; *cGC*, soluble guanylatecyclase; *cGMP*, cyclic guanosine monophosphate.

Ribeiro *et al*.^26^ has already reported that feeding efficiency is significantly correlated with salivary vasodilator activity and has demonstrated the vasodilator effect of the *R*. *prolixus* salivary homogenates using rabbit aortic rings with and without a functional endothelium. The *R*. *prolixus* salivary vasodilator is endothelium-dependent and is mediated by NO present in the SGE, which produces potent and transient relaxation in the aortic rings in *in vitro* preparations^6,43^. Based on the results of the *R*. *prolixus* SGE vasodilator activity on live hosts reported here, it is possible to propose a model showing the NO journey from the insect salivary gland to the host skin microcirculation (Fig. 9). In this model, at the feeding site (pH 7.4), NPs rapidly release NO, which could react with host molecules, probably forming S-nitroso-compounds such as the S-nitroso-albumin. In turn, according to our hypothesis, S-nitroso-albumin slowly releases NO, ensuring long-term vasodilation in the host skin.

This work shows new insights that haematophagous arthropods may use host’s molecules to modulate their salivary activities. The potential S-nitroso-albumin formation at the insects’ feeding site also opens new possibilities to investigate new salivary pharmacological activities since S-nitroso-albumin has anesthetic properties^44,45^. Hemipteran species such as *Rhodnius* and the bedbug *Cimex* may use S-nitroso-albumin to decrease the perception of the host during their blood meal. In this same context, the presence of NOS activity in salivary duct cells^46^ associated with the lack of a NO carrier in hard tick’s saliva could explain the phenomenon described in these arthropods where serum albumin from the host is recycled and secreted in their saliva^47,48^.

## Materials and Methods

### Ethics statement

All animal experiments were performed in accordance with relevant guidelines and regulations, approved by the Ethics Committee in Animal Experimentation of Federal University of Minas Gerais (CETEA/UFMG) under the protocol number 115/2011.

### Insects

A *R*. *prolixus* (Honduras) colony was reared under controlled conditions at a temperature of 28 ± 2 °C, relative humidity of 65 ± 10% and a 12/12-hour light/dark cycle. The insects were allowed to feed weekly on hamsters.

### Obtaining the salivary gland extracts

For the salivary gland extract (SGE) collections, the triatomine bugs were dissected in 0.9% saline solution using a stereo microscope, tweezers and micro-scissors. For RNA extractions, salivary glands were pooled in 1.5-mL microcentrifuge tubes containing a lysis solution. Samples were homogenised by sonication for 1 minute and then centrifuged at 12,000 g (4 °C) for 5 minutes. The supernatants were recovered and transferred to new tubes and maintained at −20 °C until the RNA extraction. For the vasodilation experiments, SG were mechanically disrupted using tweezers in three different conditions: a) in sodium acetate buffer containing 10 mM sodium acetate and 150 mM NaCl, pH 5.0, to maintain NO binding to nitrophorins at an acidic pH (this solution was named AcB-pH 5.0); b) in phosphate-buffered saline (PBS pH 7.4, 137 mM NaCl, 2.7 mM KCl, 10 mM Na_2_HPO_4_, 1.8 mM KH_2_PO) in the presence of bovine serum albumin (BSA, initial concentration 20 mg/mL), a solution that can capture most of the NO released from the NPs at an alkaline pH (this solution was named BSA-pH 7.4). After SG disruption and before injection in the mouse, the same PBS volume was added to this solution; and c) in phosphate-buffered saline (PBS), pH 7.4 - to quickly decrease unbound NO (this solution was named PBS-pH 7.4). After SG disruption and before injection in the mouse, the same volume of BSA 20 mg/mL solution was added. The two last SGE preparations that were inoculated in the mouse via intravenous or intradermal injections presented the same final composition (BSA at 10 mg/mL final concentration), differing only if the SG disruption occurred in the presence or absence of BSA. Later, all samples were centrifuged to obtain the supernatants that were injected into the mouse skin.

### RNA extraction and cDNA synthesis

RNA from the salivary glands (SG) of the *R*. *prolixus* nymphs was extracted using the Nucleospin RNA XS Kit (Macherey-Nagel) and treated with DNase according to the manufacturer’s instructions. The RNA was eluted in 20 µL of ultra-pure RNase-free water. RNA from the mouse tail epithelium was used to produce the unrelated dsRNA of mouse keratin (GenBank NM_027574), whereas RNA from different organs including antenna, midgut, integument (only the carcass, without internal organs) and head (without antenna) was obtained from the *R*. *prolixus* 5^th^ instar nymphs using the TRIzol reagent (Invitrogen) followed by a Turbo DNA-free (Applied Biosystems) treatment. RNA samples were quantified using the Nanodrop Lite spectrophotometer (Thermo-Fisher), and purity was assessed with the 260 nm/280 nm absorbance ratio (between 1.8 and 2.0). For cDNA synthesis, 0.5 µg RNA was added to 0.5 µg random hexamers (Promega) using the M-MLV reverse transcriptase system (Promega) in a 25-µL final volume. This method was used to produce cDNA for both PCR and qPCR.

### PCR and dsRNA synthesis and delivery

One microlitre of the synthesised cDNA from the salivary glands was used as a template for PCR amplification of the target genes. PCR was carried out using 200 nM specific primers conjugated to 23 bases of the T7 RNA polymerase promoter at the 5’ end (taatacgactcactatagggaga). The primers and amplicon sizes are listed in Table 1. A total of 35 cycles was carried out under the following PCR thermocycling conditions: a denaturing step at 94 °C for 40 s; an annealing step at 60 °C for 40 s and an extension step at 72 °C for 40 s. All reactions contained 200 µM deoxyribonucleotide triphosphate (dNTPs) and 1 unit of Taq DNA polymerase (Invitrogen) in a 20-µL final volume. The PCR products were used as the template for dsRNA synthesis using the MegaScript T7 Transcription Kit (Ambion) according to the manufacturer’s instructions. After synthesis, dsRNA was isopropanol-precipitated, eluted in ultrapure water and quantified by 260-nm wavelength spectrophotometry. The quality of the dsRNA products was verified by 2% agarose gel electrophoresis. dsRNA was air-dried, dissolved in sterile saline solution (0.9% NaCl) to a concentration of 10 µg/µL, and 0.5 µL of the dsRNA solution was injected into the thoracic haemolymph of each *R*. *prolixus* 3^rd^ instar nymph (under 10 days of starvation after the moult) with a microinjector (Nanoinjector, Drummond, USA). The knockdown groups were injected with 5 µg nitric oxide synthase dsRNA (dsNOS) or 5 µg nitrophorins 1–4 dsRNA, mixing 1.25 µg of each nitrophorin dsRNA (NP1, NP2, NP3 and NP4- dsNPs). The control group was injected with the same amount of keratin dsRNA (dsKer). Although dsRNA was injected in 3^rd^ instar nymphs, reductions in the mRNA levels and the phenotype alterations promoted by RNAi were assessed in 5^th^ instar nymphs^31^ (estimations of NO-loaded nitrophorins, SG aspects and feeding performance) or in adults (vasodilator activity).

**Table 1:** Primer sequences and amplicon sizes for both the PCR and qPCR experiments.

Target gene	Forward primer 5′-3′	Reverse primer: 5′-3′	Amplicon base pairs (bp)
NOS^a^	T7 + gtattcgattgccgtcaggt	T7 + gcaggacatcaaacatcgtg	332 bp
Mouse keratin^a^	T7 + ggggtctcctctctggaaac	T7 + attagcagccgtggaagaga	275 bp
NP1^a^	T7 + tttgctgcagtgggtgtaag	T7 + agttgcccgacgttacatct	380 bp
NP2^a^	T7 + gccgtgaccattctctgtct	T7 + tcacggcgcttttaactttt	548 bp
NP3^a^	T7 + tggaaccgtactcagctttg	T7 + gcgtttttaaccgttgcact	566 bp
NP4^a^	T7 + cgtagccattctgtgcctgt	T7 + gcagatacggcgcttttaac	547 bp
α-tubulin^b^	tttcctcgatcactgcttcc	cggaaataactggggcataa	129 bp
NOS^b^	acgaagattggcgatactgg	gcgtacagagatggtgcaga	151 bp
NP2^b^	catatgtttgcgggaaggat	atcgtactggcacccaagat	163 bp

^a^PCR primers (used for dsRNA template). ^b^qPCR primers.

### Real-time quantitative polymerase chain reaction (qPCR)

Quantitative PCR reactions were performed using the StepOne Plus real-time quantitative PCR system (Applied Biosystems) for the following evaluations: *R*. *prolixus* NOS expression in different organs; NOS and NP2 expression in the salivary glands of insects under different feeding conditions; and confirmation of NOS RNAi knockdown in the salivary glands. Each reaction was run in duplicate and contained 20 ng of cDNA, a 300-nM final concentration of primers and 7.5 µL of the Power SYBR Green PCR Master Mix (Applied Biosystems) in a 15-µL final volume. The qPCR primer sequences are listed in Table 1. Amplification conditions were 95 °C for 10 min, 40 cycles of 95 °C for 15 s and 60 °C for 1 min. A negative control without reverse transcriptase and a negative control without the template were included for each primer set to confirm the absence of genomic DNA and to assess primer-dimer formation or other contamination in the reactions, respectively. The qPCR efficiency was determined for each gene using the slope value in the following equation: Efficiency = (10^[−1/slope]^ − 1) × 100; the slope was determined with serial cDNA dilutions (100 ng, 10 ng, 1 ng, 0.1 ng, and 0.01 ng) in a template linear regression analysis. To ensure that only single products were amplified, a melting curve analysis was performed. The relative amount of the gene product in each sample was determined using the 2^−ΔΔCt^ method^49^, with α-tubulin as the reference gene^50^.

### Estimation of haemeproteins and NO-loaded nitrophorins in the salivary glands

The amount of nitric oxide inside the SG was indirectly estimated by quantification of NO-loaded nitrophorins or nitrophorins in the absence of NO. The SG pairs from the individual bugs were collected and disrupted in 200 µL of AcB-pH 5.0. At this pH, NO–nitrophorins ligation remained stable. After gland homogenisations by sonication, the solution was centrifuged at 4 °C for 10 min at 14,000 g. The supernatant was recovered and analysed using a spectrophotometer (Shimadzu UV-1650 PC) at wavelengths of 422 nm and 404 nm. The absorbance at 280 nm was measured as an indication of the total protein content, and the sum of the 404 nm + 422 nm absorbances was used as an indirect indication of the total haemeprotein content. The absolute absorbance values were compared between the dsNOS knockdown and dsKer control group. The 422/404 absorbance ratio was used to estimate the degree of NO loading of the nitrophorins, based on the differential spectral profiles of the NO-loaded (422 nm) and unloaded nitrophorins (404 nm)^9,30^.

### Feeding performance on live hosts

Feeding performance of the *R*. *prolixus* 5^th^ instar nymphs injected with dsKer or dsNOS during the third instar (approximately 60 days before the feeding performance evaluation) was assessed from insects that fed on the dorsal surfaces of anaesthetised rats (*Rattus norvegicus*). For the assays, rats weighing approximately 150 g were anaesthetised with 150 mg/kg ketamine (Cristalia) and 10 mg/kg xylazine (Bayer) by intraperitoneal injection. For the experiments, 10 insects from the same group were placed in contact with the shaved dorsal surface of the rodent for 25 min and allowed to feed simultaneously on the host. Each insect was weighed before and immediately after feeding to calculate weight gain. To facilitate identification of the individual insects, different colours of a non-toxic paint were applied (Color Make) to the dorsal surface of each insect. To restrict the feeding site, an arena (plastic container of 11.5 × 8.0 × 2.5 cm) with an oval shaved area (with an approximate area of 20 cm^2^) was placed on the rat dorsal surface. Throughout the experiment, the temperature of the host was monitored using a rectal sensor and maintained at 37 °C with a heating pad (Fine Science Tools In). The feeding period was recorded using a camera (Olympus U-PMTVC) for later analysis. The recorded movies were used to calculate the contact time (time that the mouth of the insect remained in contact with the host). The ingestion rate was calculated by dividing the weight gain by the contact time, and the number of bites to the host was also evaluated.

### Vasodilator activity assays by intravital microscopy

Forty- to sixty-day-old hairless mice were anaesthetised by an intraperitoneal injection of 150 mg/kg ketamine and 10 mg/kg xylazine. The experiments were performed on the convex dorsal surface of the hairless mouse ear, which was fixed on a glass support with an adhesive double-sided tape. After ear fixation, a resting time of 30 min was allowed for stabilisation of the microcirculation vessels before the start of the experiments. Throughout the experiments, the mouse temperature was maintained at 34 °C with a heating pad (Fine Science Tools Inc., Canada). Hairless mouse ear vascularisation usually originates from three pairs of arterioles and venules on the ear base, and four blood vessel subdivisions (or orders) are observed from these three pairs^51^. All experiments were performed in the second order venules of the mouse ear to avoid large differences in the diameters of the analysed vessels.

To compare the SGE vasodilator activity of the knockdown (dsNOS or dsNPs) and control (dsKer) insects, intravital microscopy of the mouse ear was performed with intradermal injections into the mouse ear skin or with intravenous injections into the mouse tail vein. In both experiments, we used salivary gland extracts (SGE) from *R*. *prolixus* that were injected with dsRNA during the third instar and maintained under starvation for 10 days after reach the adult stage.

For the intravenous injections, one pair of SGs was disrupted in 70 µL of AcB-pH 5.0 (sodium acetate buffer: 10 mM sodium acetate and 150 mM NaCl, pH 5.0), or three salivary gland pairs were disrupted in 70 µL of BSA-pH 7.4 (10 mg/mL BSA final concentration in PBS, pH 7.4). The initial bovine serum albumin concentration (BSA- 20 mg/mL) used in salivary gland disruption was similar to the albumin concentration found in murine interstice^52^. Afterwards, the samples were quickly centrifuged (700 g, 20 s) and supernatants were injected into the mouse tail vein. For intradermal injections, one salivary gland pair was disrupted in 5 µL of BSA-pH 7.4. The samples were quickly centrifuged, and 32 nL of the supernatant was injected into the space between the two vessels of the mouse ear.

The vessel diameters of the mouse ear skin were monitored immediately before and 30 min after the injections using an optical microscope (Leica DM500). Photographs of the skin microcirculation were obtained one minute before the injection, immediately after the injection and throughout a 30 min period after the injections. Images (one photo per minute, 720 × 400 pixels) were captured with a digital camera (Canon EOS 600D). A fixed area covering the vessel was selected to be subsequently analysed. The estimation of the area occupied by the vessel was performed using the ImageJ software^53,54^ using the product of the area and mean grey value (integrated density). The integrated density values corresponding to the area occupied by blood were incorporated into the GraphPad Prism 5 software for further analyses. To facilitate the comparisons, the venule diameter value before the injection was arbitrarily set to 1, whereas for the intradermal injections, the maximum dilation in each experiment was arbitrarily set to 1.

### Statistical analyses

Data are represented as the mean ± S.E. for values with normal distribution or as the median ± interquartile range for values with non-normal distributions. The Kolmogorov–Smirnov test was used to identify variables with normal distributions which were then compared by t-test or by one-way or two-way ANOVA with post-hoc comparisons made using Bonferroni’s test. The non-parametric Mann–Whitney test or Kruskal–Wallis test followed by Dunn’s test was used for variables with non-normal distributions. Data were analysed and plotted using the GraphPad Prism 5.0 software, with p < 0.05 as the accepted level of significance.

The datasets generated during and/or analysed during the current study are available from the corresponding author on reasonable request.
